# Olive Leaf Extract Supplementation Improves Postmenopausal Symptoms: A Randomized, Double-Blind, Placebo-Controlled Parallel Study on Postmenopausal Women

**DOI:** 10.3390/nu16223879

**Published:** 2024-11-14

**Authors:** Maria Imperatrice, Anissa Lasfar, Colin A. J. van Kalkeren, Freddy Troost

**Affiliations:** 1 BioActor BV, Gaetano Martinolaan 50, 6229 GS Maastricht, The Netherlands; anissa.lasfar@maastrichtuniversity.nl; 2 Department of Nutrition and Movement Sciences, Institute of Nutrition and Translational Research in Metabolism (NUTRIM), Maastricht University, 6200 MD Maastricht, The Netherlands; 3 Department of Human Biology, Institute of Nutrition and Translational Research in Metabolism (NUTRIM), Maastricht University, 6200 MD Maastricht, The Netherlands; c.vankalkeren@maastrichtuniversity.nl (C.A.J.v.K.); f.troost@maastrichtuniversity.nl (F.T.); 4 Food Innovation and Health, Centre for Healthy Eating and Food Innovation, Maastricht University, 5911 AA Venlo, The Netherlands

**Keywords:** olive leaf extract, oleuropein, menopause, women’s health, human

## Abstract

Menopause negatively impacts women’s health. Objectives: The aim of this study was to investigate whether an olive leaf extract (OLE) improves postmenopausal symptoms, body composition, handgrip strength and blood lipid profile in postmenopausal women. In a randomized, double-blinded parallel study design, 60 healthy postmenopausal women aged 47–70 years received either OLE (250 mg/day) or placebo supplementation for 12 weeks. Postmenopausal symptoms were assessed with the Menopause-Specific Quality of Life Questionnaire (MENQoL), the Hot Flash Interference scale (HFI), and body composition and bone mineral density (BMD) with a DXA scan; the lipid profile was measured in the blood serum. After six and twelve weeks of OLE supplementation, the overall MENQoL score significantly improved (estimated mean difference [95% CI]: −0.2 [−0.4−0.2], *p* = 0.027) compared to the placebo. A significant improvement (+0.017 [0.003, 0.030], *p* = 0.019) was recorded in the BMD in the right arm in the OLE group compared to the placebo. The intervention did not affect other body composition outcomes. TG concentrations and the TG/HDL-C ratio were significantly decreased (−0.1 [−0.2, 0.0], *p* = 0.010; −0.1 [−0.2, −0.0], *p* = 0.029, respectively) in the OLE group compared to the placebo. Twelve weeks of daily OLE supplementation improved postmenopausal symptoms. Further studies are needed to elucidate the mechanisms underlying the observed effects.

## 1. Introduction

Menopause starts 12 months after the last menstrual period and marks the end of fertility [1]. The majority of women, in our modern, economically developed society live for at least 20–40 years in a postmenopausal state [2]. The end of a woman’s reproductive life is induced by the gradual or sudden cessation of estradiol and progesterone secretion by the ovaries, which impacts various tissues, including skeletal muscle and skin [3]. During menopause, women may experience physical, emotional, and urogenital symptoms, with a significant impact on their health [1,2]. These include hot flashes, night sweats, sleep disruption, fatigue, concentration problems, frequent urination, depression and anxiety, mood swings, irritability, and a decrease in sexual desire [4]. Treatment options to alleviate menopausal symptoms, include hormone replacement therapy (HRT). Such treatments are associated with side effects. The Million Women Study highlighted the correlation between estrogen–progestogen replacement therapy and increased the incidence of breast cancer [5,6]. Moreover, prolonged exposure to excess estrogen leads to an increased risk of estrogen-dependent diseases, such as endometrial cancer [7]. Furthermore, HRT has been correlated to a higher rate of depressive symptoms in Dutch menopausal women [8]. Therefore, there is increasing interest in more natural alternative therapies to counteract postmenopausal symptoms with substances that mimic estrogen’s effects without causing its associated negative side effects [9].

Phytoestrogens are plant-derived dietary compounds that are structurally similar to 17-β-oestradiol (E2), the primary female sex hormone [10]. Due to their structural similarity, phytoestrogens can bind to estrogen receptors, producing estrogenic effects. Oleuropein is the most abundant polyphenol found in olive tree leaves (*Olea europaea*) [11]. It belongs to the class of secoiridoids, and its hydrolysis produces oleuropein aglycone, elenolic acid, hydroxytyrosol (HT), and glucose [12]. Interestingly, oleuropein and HT both contain an aromatic ring resembling that of oestradiol, leading to the hypothesis that these compounds may compete with estrogen for receptor-binding sites [11]. However, the effect of olive leave extract (OLE) on postmenopausal symptoms remains to be investigated.

During menopause, the reduction in estrogen is associated with increased risk of osteoporosis, a disease that reduces bone strength and density, which is associated with an increased risk of bone fractures. It was previously reported that twelve months of OLE consumption standardized for oleuropein had a protective effect on the loss of bone mineral density (BMD) [13]. Moreover, six months of OLE supplementation was effective in relieving knee joint pain and enhancing mobility in subjects aged 55 and above with articular pain [14].

Another symptom of ageing, which may develop during and after menopause, is the reduction in muscle mass called sarcopenia. Physiological and morphological changes in skeletal muscle caused by ageing are marked by a significant infiltration of adipose tissues into the skeletal muscle and an overall decline in the quantity and size of muscle fibres [15], which results from an increase in muscle proteolysis and a decrease in protein synthesis [16]. Polyphenols, especially flavonoids, show the potential for counteracting muscle atrophy through the stimulation of protein synthesis and inhibition of protein degradation mechanisms [17,18,19]. When tested in old rats, OLE supplementation successfully attenuates the ageing-induced decrease in protein content in skeletal muscle and increases insulin sensitivity in adipose tissue and skeletal muscle [20].

Finally, during menopause, the decreased level of estrogen leads to lipid metabolism dysregulation. Estrogens play a protective role in the cardiovascular system, being primarily produced in the ovaries through a process that utilizes low-density lipoprotein cholesterol (LDL-C) as a substrate. However, when LDL-C is not used to synthesize estrogen anymore, its circulating concentration increases [21]. LDL contributes to the formation of plaque deposits that can compromise the arteries, thereby hampering their compliance. Moreover, it has been shown that triglyceride (TG) levels peak in women exhibiting menopausal transition compared to premenopausal women [22]. For these reasons, during menopause, there is an increased risk of developing cardiovascular diseases (CVDs) such as coronary heart disease, myocardial infarction, and stroke [23]. Previous studies involving human subjects with elevated blood pressure or overweight suggest that supplementation with OLE can positively impact CVD risk factors [24,25,26].

The aim of this randomized, double-blind, placebo-controlled, parallel study was to determine the effect of 12 weeks of 250 mg/day of OLE supplementation on menopause-related quality of life, body composition, muscle strength, and blood lipid profile in postmenopausal women (45–70 y) compared to control (cellulose).

## 2. Materials and Methods

### 2.1. Study Population

Healthy postmenopausal (amenorrhea over 12 months) women (aged 47–70 years) with a BMI <35 kg/m^2^ were recruited through local advertisements on newspapers and social media. Interested participants were invited to attend a screening visit to determine their eligibility. Exclusion criteria were the use of HRT (estrogenic or progestogenic) 3 months before the start of the study; the use of antibiotics and/or supplements (e.g., probiotics or supplements containing vitamins, minerals, antioxidants, and isoflavones) within 3 months before the start of the study (calcium and Vit D were allowed with a constant daily dosage intake); smoking and abuse of alcohol (>20 alcoholic units/week) or drugs; history of breast cancer; presence of surgical metal elements in the body; and gastric bypass.

All participants gave written informed consent before data collection. This study was approved by the Institutional Review Board Medical Ethics Assessment Committee of University Hospital Maastricht and Maastricht University (METC azM/UM) and performed at the University of Maastricht between January 2023 and May 2024 in accordance with the 1964 Declaration of Helsinki and its later amendments (latest amendment Fortaleza, Brazil, 2013). This study was registered online at ClinicalTrials.gov as NCT05744453.

### 2.2. Study Design

A randomized, double-blinded, placebo-controlled, parallel study was performed. Eligible participants were randomly allocated to receive OLE or placebo (cellulose) supplementation for 12 weeks. The randomization schedule was generated by a computer and carried out by an independent researcher. All participants and investigators remained blinded to the treatment until all analyses were completed.

**Figure 1:**
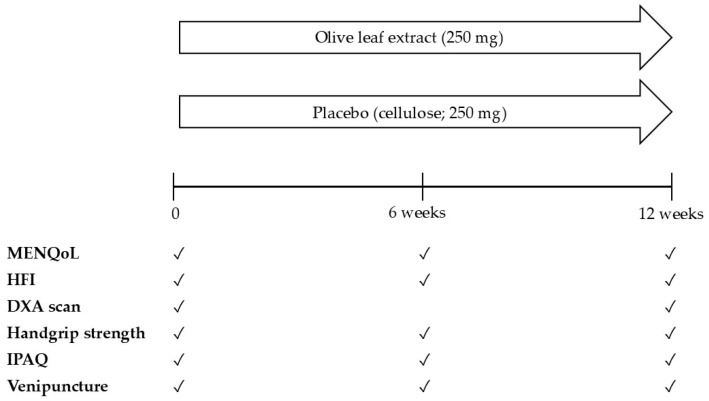
Study design. MENQoL: Menopause-Specific Quality of Life; HFI: Hot Flash Interference scale; DXA: Dual-energy X-ray absorptiometry; IPAQ: International Physical Activity Questionnaire.

Eligible participants completed three study visits (at baseline, and after 6 and 12 weeks). All study visits were performed in the morning in a fasted state in temperature-controlled rooms at the Metabolic Research Unit Maastricht (MRUM). Subjects were asked to maintain their dietary habits during the entire study period and to avoid strenuous physical activity and alcohol on the day prior to each test day. During all test days, fasted blood samples were collected from an antecubital vein. Questionnaires were completed, and handgrip strength was measured. DXA was performed only at baseline and after 12 weeks. The study design is depicted in Figure 1.

### 2.3. Intervention

The study product was a commercially available OLE from Spanish Manzanilla olive leaves (Bonolive^®^; Maastricht, The Netherlands) standardized for its oleuropein (40%) content. A daily dose of 250 mg OLE was provided in one capsule containing 100 mg oleuropein per day. This dosage is based on a previous clinical study assessing the effect of Bonolive^®^ on lipid profiles and bone health in postmenopausal women [13]. The control was cellulose, 250 mg in one capsule. Participants were instructed to consume one capsule per day with a glass of water each morning at breakfast and record the daily intake, along with any deviations, in a supplementation logbook.

#### 2.3.1. Postmenopausal Symptoms

Postmenopausal symptoms were assessed with the Menopause-Specific QoL Questionnaire (MENQoL) (Mapi Research Trust, Lyon, France) [27,28]. The MENQOL is a 29-item questionnaire designed to measure four domains of menopausal symptoms (vasomotor, psychosocial, physical, and sexual) in the past month. Participants were asked whether they had experienced any of the 29 symptoms within the past month and if the answer was yes (they had experienced the specific symptom), they were further asked to rate how bothersome each symptom was on a 7-point Likert scale (0 = not at all bothered; 6 = extremely bothered). The 29 items are combined into four domains: vasomotor (three items), psychosocial (seven items), physical (sixteen items), and sexual (three items). Each domain score ranges from 1 to 8 and is calculated as the mean of the converted item scores forming that domain. Higher scores indicate more severe menopausal symptoms and a lower quality of life in postmenopausal women.

Hot flashes were measured via the Hot Flash Interference scale (HFI) (Mapi Research Trust, Lyon, France) [29], a three-item questionnaire evaluating hot flashes’ interference with sleep, mood, and concentration in the past two weeks. HFI scores range from mild (0 to 3.9) to moderate (4 to 6.9) to severe (7 to 10) symptoms.

#### 2.3.2. Body Composition

Whole-body and regional body composition was determined using Dual-energy X-ray absorptiometry (DXA) (Horizon^®^ DXA System, Hologic, Marlborough, MA, USA) [30]. DXA is regarded as an effective technique used to assess muscle mass and body composition in research and clinical settings [31]. Body composition parameters, including regional (left arm, right arm, trunk, left leg, right leg) and total body fat-free mass (FFM), fat mass (FM), bone mineral density (BMC), visceral abdominal tissue (VAT), and appendicular lean mass (ALM) were obtained. ALM alone, or scaled to height squared (ALM index, ALMI), is the most commonly used metric to approximate muscle mass in sarcopenia research. DXA was conducted at baseline and after 12 weeks.

#### 2.3.3. Handgrip Strength Measurements

Isometric handgrip strength was chosen as a reliable and valid surrogate measure of overall muscular strength [32]. With the use of a calibrated hand dynamometer (Model SH5001, Saehan, Seoul, Republic of Korea), handgrip strength was measured on both hands. All measures were performed while the participant was seated in an upright position, with the arm of the measured hand parallel to the body. Before each measurement, the width of the dynamometer’s handle was adjusted to fit each participant’s hand size to ensure that the middle phalanges rested on the inner handle. Participants were then instructed to exert maximal force. Each participant performed three consecutive test measurements, alternating between hands, with ~60 s rest intervals between each measure. All measures were recorded to the nearest 0.5 kg. The highest scores were used for the analysis.

#### 2.3.4. Physical Activity

The long version of the self-administered International Physical Activity Questionnaire (IPAQ-L) was utilized to assess participants’ physical activity levels [33]. The IPAQ-L measures the duration, frequency, and intensity of physical activities across various domains, including recreation/leisure, work, transportation, and household/gardening tasks. Data obtained from the IPAQ-L were used to estimate the total physical activity per week (MET-min/wk.^−1^). Data on total sitting time were also collected from the IPAQ-L. Participants were asked to report the time spent sitting at work, at home, and during leisure activities over the previous 7 days.

#### 2.3.5. Biochemical Analyses

Fasted blood samples were taken on each test day and collected in serum separator tubes (BD Vacutainer, Franklin Lakes, NJ, USA). After blood collection, serum tubes were stored at room temperature for at least 30 min until complete coagulation and centrifuged within one hour (20 °C, 1300× *g*, 10 min). After centrifugation, serum aliquots were snap-frozen and stored at −80 °C until analyses. Blood lipid concentrations (total cholesterol, HDL-C, and TG) were determined using an (homogeneous) enzymatic colorimetric test (Cobas Pure c303, ROCHE Diagnostics GmbH, Basel, Switzerland). The blood analyses were performed at the Laboratory of Central Diagnostics at the MUMC+ hospital in Maastricht, the Netherlands. The LDL-C was calculated with the Friedwald formula [34].

### 2.4. Statistical Analyses

An expected effect size of 0.91 was determined, based on the change in appendicular fat-free mass mean outcome values in the intervention group compared to the control, in the study of Aubertin-Leheudre et al. [35]. With a power of 0.90, a two-sided alpha of 0.05, and a standardized effect size of 0.91, a sample size of at least 54 participants was required. Taking into account an expected drop-out rate during the intervention of 15%, at least 64 subjects had to be included. Statistical analyses were performed using IBM SPSS Statistics (26.0, IBM Corporation, Armonk, NY, USA).

For DXA outcomes, intention-to-treat analyses were performed with linear mixed models with treatment as the fixed factor, participant as the random factor, and baseline values as the covariate. Additionally, BMI was added to the model as a potential confounder. For handgrip strength, blood outcomes, MENQoL, HFI, and IPAQ questionnaire statistical analyses were performed using intention-to-treat analyses with linear mixed models including treatment and time as fixed factors, time × intervention as the interaction term, baseline values as covariates, and participants and intercepts as random factors. The two-way interaction was omitted from the model if not significant. Data were reported as unadjusted means ± SDs, unless stated otherwise. For all analyses, two-sided *p*-values ≤ 0.05 were considered statistically significant.

## 3. Results

In total, 77 participants were screened for eligibility, of which 62 were included in the current study (Figure S1. CONSORT flow diagram). Two participants allocated to the OLE group discontinued the intervention: one after the baseline measurements took place because of health-related reasons unrelated to the study; one after 6 weeks because of antibiotics intake. There were no dropouts in the control group. Baseline characteristics of the study population are described in Table 1. The study product was well tolerated with no serious adverse events or protocol deviations reported.

**Figure 2:**
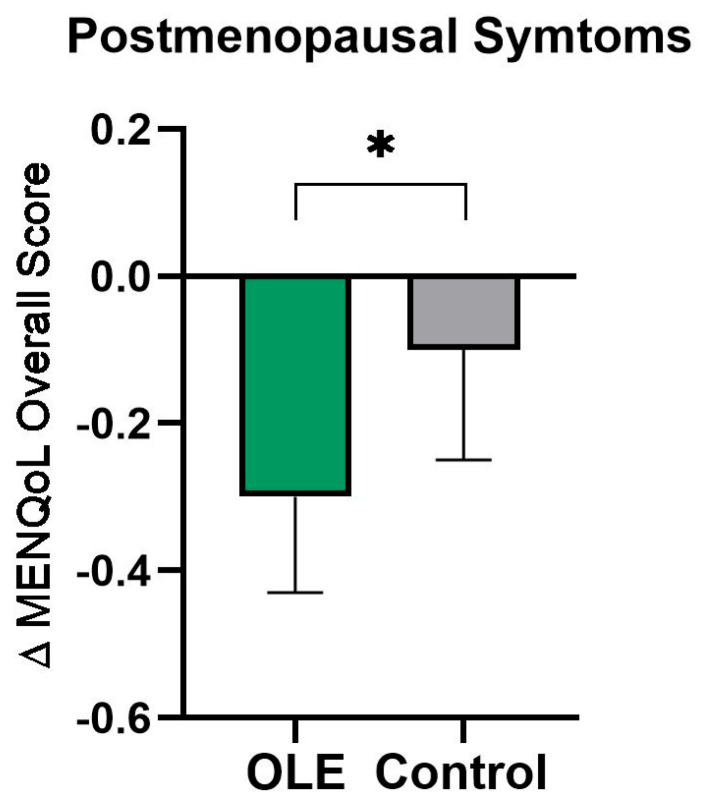
The change in the MENQoL overall score following OLE or control supplementation. Data are presented as mean change from baseline ± SEM. Analyses were performed with a linear mixed model using time and treatment as a fixed factor and participant as a random factor. * indicates *p* ≤ 0.05. Abbreviations: MENQoL: Menopause Quality of Life questionnaire; OLE: olive leaf extract.

The results of the MENQoL Overall Score are displayed in Table 2. Vasomotor, psychosocial, physical, and sexual domain scores taken singularly were not significantly different between OLE and placebo groups. However, a trend for time × treatment interaction was found in the physical domain (*p* = 0.058). Moreover, the overall MENQoL score was significantly improved (*p* = 0.027) after OLE supplementation compared to placebo (Figure 2; Table 2).

No significant treatment effects were recorded for the HFI (Table 3) and IPAQ questionnaires (see Table S1).

**Figure 3:**
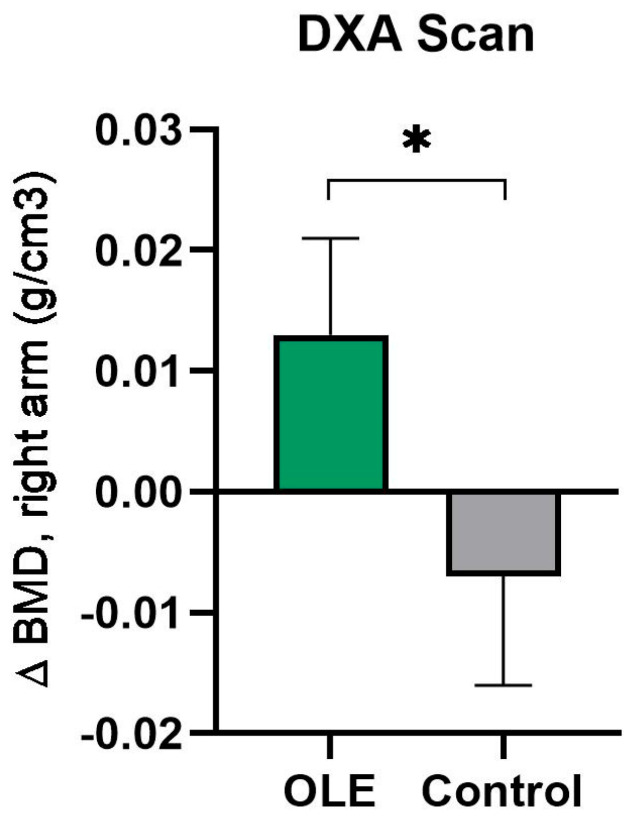
The change in BMD in the right arm following OLE or control supplementation. Data are presented as mean change from baseline ± SEM. Analyses were performed with a linear mixed model using time and treatment as a fixed factor and participant as a random factor. * indicates *p* ≤ 0.05. Abbreviations: BMD: bone mineral density; DXA: Dual-energy X-ray absorptiometry; OLE: olive leaf extract.

In Table 4, the effects of OLE supplementation on body composition and bone mineral density measured via a DXA scan are shown. No significant effects on fat mass (i.e., total body fat (%), total body fat mass (g), estimated VAT) or fat-free mass (i.e., ALMindex, Lean/Height^2^) were observed. Bone mineral density (BMD) in the right arm significantly increased in the OLE group after 12 weeks compared to placebo (*p* = 0.019) (Figure 3). Moreover, a trend for treatment effect on BMD right ribs was found (*p* = 0.055). No significant effects on other aspects of BMD were observed (see Table S2).

Results of the handgrip strength test are reported in Table 5. For the higher strength scores in both hands, no significant effects were observed. However, a trend for a time × treatment interaction on the higher strength score in the right hand was found (*p* = 0.055).

**Figure 4:**
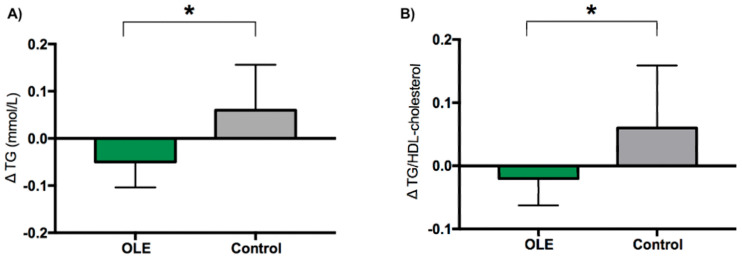
The change in serum triglycerides (**A**) and the triglyceride/HDL cholesterol ratio (**B**) following OLE or control supplementation. Data are presented as mean change from baseline ± SEM. Analyses were performed with a linear mixed model using time and treatment as a fixed factor and participant as a random factor. * indicates *p* ≤ 0.05. Abbreviations: OLE: olive leaf extract; TG: triglycerides.

The results of the blood lipids analyses are reported in Table 6. TG concentrations and the TG/HDL-C ratio were significantly decreased (*p* = 0.010; *p* = 0.029, respectively) in the OLE supplementation group compared to placebo. No significant changes were recorded in the total cholesterol, HDL-C, and LDL-C concentration outcomes. The TC/HDL-C ratio was also not significantly different between OLE and placebo (Figure 4).

## 4. Discussion

In this randomized, controlled, parallel trial involving postmenopausal women, the effects of OLE on postmenopausal symptoms were investigated for the first time. Six weeks of OLE consumption significantly improved the quality of life of women experiencing postmenopausal symptoms, an improvement that remained stable over 12 weeks of supplementation. The MENQoL overall score includes a multitude of negative symptoms that women experience during the menopause period, such as a decrease in sexual desire, difficulty sleeping, poor memory, feelings of dissatisfaction, anxiety, decrease in strength and energy, frequent urination, sweating, and hot flashes, among others.

Although the effects of OLE on postmenopausal symptoms have not been previously studied, it has been hypothesized that these effects may be attributed to the chemical similarity between OLE and phytoestrogens, and consequently, to their similarity with estrogenic compounds. Multiple studies have investigated the effects of phytoestrogens on menopausal symptoms [36,37]; however, the role of phytoestrogens in alleviating postmenopausal symptoms remains under investigation [38]. The main concern relates to the proposed dualistic mechanism of action, which involves the capacity of phytoestrogen to selectively bind specific receptors, namely estrogen receptor α (Erα) and estrogen receptor β (Erβ). The activation of Erα enhances cell proliferation, necessary for growth and tissue maintenance in the breast and uterus. Erα is also linked to breast cancer cell proliferation. Erβ, instead, has been shown to counteract the Erα-mediated stimulation of cell proliferation [39]. Phytoestrogens show a higher binding preference for Erβ than for Erα [40]. Therefore, when compared to estrogen and HRT, phytoestrogen exposure has been related to reduced risk of side effects.

In vitro studies assessing the molecular mechanism behind the OLE effect on ER in postmenopausal women have not been performed yet. Park et al. [41] examined the effect of oleuropein in reproductive-aged women with endometriosis, an estrogen-dependent gynecological disorder that negatively affects the pregnancy rate. When tested in human endometrial cells, oleuropein selectively inhibited Erβ, but not Erα activity in vitro. Erβ, in conjunction with high oestradiol levels, is essential in the development of endometriosis in reproductive-aged women. Moreover, in the same study, the oral intake of oleuropein improved the fertility of mice with endometriosis. These results suggest oleuropein’s potential as a novel nutraceutical product for non-hormonal therapy in premenopausal women. To date, similar studies on oleuropein in postmenopausal women have not yet been conducted. Important to consider is that hormonal status and age may influence phenolic bioavailability [42]. Garcia-Villalba et al. [43] demonstrated that OLE exhibits higher bioavailability in postmenopausal women compared to premenopausal women following oral intake. Since the underlying mechanisms of OLE on postmenopausal symptoms remain unclear, further in vitro studies in humans are necessary to elucidate the improvements in postmenopausal symptoms observed in our study.

Contrary to our hypothesis, no significant change in body composition outcomes (specifically related to FM or FFM) were observed after 12 weeks of OLE supplementation. Our findings are in contrast with those of Haidari et al. [44], who found that 8 weeks of OLE supplementation (250 mg/day) combined with a caloric-restrictive diet, significantly decreased fat mass in obese women, compared to diet only. Additionally, Binou et al. [45] reported that 12 weeks of dietary intervention involving daily consumption of bread enriched with hydroxytyrosol (32.5 mg/day), an oleuropein metabolite, resulted in significant body fat mass reduction in subjects with overweight/obesity and type 2 diabetes mellitus. These findings suggest that 12 weeks of OLE supplementation may not be sufficient to induce changes in body composition parameters without concurrent caloric restriction or dietary intervention. OLE is known for its protective effect on the loss of BMD. In our study, we observed an improvement in BMD in the right arm after 12 weeks. Moreover, a trend for increased BMD in the right ribs was observed. These findings are consistent with previous research, such as the study by Filip et al. [13], which demonstrated that three months of OLE consumption increased serum total osteocalcin, a marker of bone turnover, in postmenopausal women with osteopenia. Moreover, Puel et al. [46] reported that 100 days of oleuropein intake provided protective effects on bone mass in an experimental rat model of bone loss. While our results align with these earlier studies, they suggest that a longer intervention period may be necessary to detect more pronounced changes in BMD in humans.

In the study by Terzic et al., phytoestrogens improved serum lipid profile in postmenopausal women [47]. In the study by Filip et al., 12 months of OLE supplementation improved serum lipid profile (TC, LDL-C, and TG) in postmenopausal women with osteopenia [13]. Other studies with shorter intervention (4–8 weeks) periods and different study populations have also shown improved lipid profiles after OLE supplementation [26,48,49]. Likewise, our results show that TG levels were affected by OLE supplementation during an intervention period of 12 weeks. Additionally, the TG/HDL ratio, a marker for cardiovascular disease (CVD) risk, was significantly lower in the intervention group compared to control after OLE supplementation. Estrogen has cardioprotective effects; however, during menopause, estrogen levels decline, which is associated with lipid dysregulation (e.g., reduced HDL-C) [50]. This could result in the accumulation of fat in the arteries leading to atherosclerosis and arterial occlusion, increasing the risk of CVD. Our results show that OLE improves lipid dysregulation and might thereby decrease the risk of CVD. This might be explained by the phytoestrogenic effect of oleuropein. Our study contributes to the understanding of the duration needed for OLE to positively influence lipid profiles in postmenopausal women. The short-term beneficial effect of OLE on TG levels reported in our study could enhance patient compliance with future supplementation therapies. Further research should also investigate the effect of OLE on the apolipoprotein AI (ApoA-I), a biomarker that better describes the antiatherogenic role of HDL particles or HDL-cholesterol [51].

Previous studies suggest that a potential mechanism for oleuropein’s protective effects on bone mass loss and improvement in lipid profiles is its ability to enhance osteoblastogenesis (the formation of bone cells) while inhibiting adipogenesis (the formation of adipose tissue cells) [52]. Notably, both osteoblasts and adipocytes originate from a common progenitor, mesenchymal stem cells (MSCs), which are regulated by oleuropein. These findings imply that oleuropein may favour the formation of osteoblasts over adipocytes, potentially contributing to the prevention of age-related bone loss and osteoporosis, while also reducing lipid levels [52]. However, the exact mechanism is not known. Further in vitro studies are needed to elucidate the precise mechanisms underlying these effects. Additionally, it is worth noting that another polyphenol, genistein, has been shown to exert opposing effects on osteogenesis and adipogenesis by concurrently activating two distinct transcription factors, estrogen receptors (ERs), and peroxisome proliferator-activated receptor γ (PPARγ) [53]. The potential involvement of ERs may further elucidate oleuropein’s impact on the aforementioned postmenopausal symptoms.

The findings of this study have to be interpreted while considering some limitations. We did not take into consideration including only women experiencing moderate to severe vasomotor symptoms (e.g., hot flashes). The mean of the vasomotor symptoms in our study population ranged from mild to moderate. Therefore, it is less likely to observe an improvement in the HFI questionnaire, which was the case. Despite this, we observed a significant improvement in the complete spectrum of the postmenopausal symptoms (MENQoL overall scores) when comparing the OLE group to the placebo. For a follow-up study design, we suggest including participants experiencing moderate-to-severe vasomotor symptoms. A second limitation of this study, which may have influenced the muscle mass assessment, is the technique chosen to measure body composition. A recent study by Fuchs et al. [54] demonstrated that DXA is less sensitive than CT and MRI for assessing age-related differences in muscle mass. Therefore, in future studies, a more sensitive technique for assessing body composition may be preferable to accurately detect changes in muscle mass in postmenopausal women.

A notable strength of this study is its exploration of the effect of OLE on postmenopausal symptoms using the validated MENQoL questionnaire. This comprehensive tool assesses a broad range of menopausal symptoms beyond the commonly acknowledged hot flashes, providing a more nuanced understanding of women’s experiences during menopause. Our findings pave the way for further research into the impact of non-hormonal interventions on the quality of life in postmenopausal women, thus contributing valuable insights to the scientific community.

## 5. Conclusions

In conclusion, OLE supplementation improved postmenopausal symptoms, BMD, serum TG, and the TG/HDL ratio in postmenopausal women. Therefore, OLE can be evaluated as an effective nutritional strategy to help women navigate the postmenopausal years being less affected by its negative symptoms. Postmenopausal health is a crucial aspect in women’s successful ageing, underscoring the need for further research to clarify the mechanisms driving observed health outcomes. Gaining a deeper understanding of the cellular mechanisms involved, i.e., investigating the effects of oleuropein on estrogen receptors, will increase scientific knowledge and understanding on the topic and support more effective interventions for the postmenopausal population.

## Figures and Tables

**Table 1:** Baseline characteristics of the OLE and control group ^1^.

	Total Population (*n* = 60)	OLE (*n* = 29)	Control (*n* = 31)
Age (years)	59.2 ± 5.8	58.4 ± 6.2	59.8 ± 5.4
Height (cm)	165 ± 6.7	165.5 ± 7.2	165.2 ± 6.3
Weight (kg)	69.7 ± 9.6	68.5 ± 8.6	70.8 ± 10.5
BMI (kg/m^2^)	25.4 ± 2.8	24.9 ± 2.1	25.9 ± 3.4

^1^ Values are presented as mean ± SD. Abbreviations: BMI: Body Mass Index; OLE: olive leaf extract.

**Table 2:** MENQoL questionnaire scores following the OLE and control group in postmenopausal women ^1^.

	OLE			Control			Time × Treatment Interaction	Treatment Effect	Estimated Mean Difference [95% CI]
	Baseline	After 6 Weeks	After 12 Weeks	Baseline	After 6 Weeks	After 12 Weeks			
Vasomotor Score	2.2 ± 1.4	2.2 ± 1.6	2.2 ± 1.5	2.0 ± 1.3	2.2 ± 1.4	2.3 ± 1.7	*p* = 0.607	*p* = 0.489	−0.1 [−0.6 + 0.3]
Psychosocial Score	1.8 ± 0.9	1.6 ± 0.9	1.6 ± 1.1	2.0 ± 1.2	1.7 ± 1.0	1.7 ± 1.1	*p* = 0.753	*p* = 0.905	−0.0 [−0.3 + 0.3]
Physical Score	2.1 ± 0.9	1.7 ± 0.6	1.8 ± 0.9	1.9 ± 0.8	1.8 ± 0.8	1.7 ± 0.7	*p* = 0.058	*p* = 0.195	−0.1 [−0.4 + 0.0]
Sexual Score	2.0 ± 1.5	1.4 ± 0.9	1.3 ± 0.7	1.6 ± 1.3	1.6 ± 1.1	1.5 ± 1.1	*p* = 0.808	*p* = 0.116	−0.3 [−0.6 + 0.7]
Overall Score	2.0 ± 0.7	1.7 ± 0.6	1.7 ± 0.8	1.9 ± 0.9	1.8 ± 0.8	1.8 ± 0.9	*p* = 0.628	*p* = 0.027 *	−0.2 [−0.4 − 0.2]

^1^ Values are presented as mean ± SD. *n* = 60. Differences between OLE and control were calculated with a linear mixed model analysis with a random intercept. Time × treatment was used as a fixed factor and participant as a random factor. If the interaction term was not significant, it was removed from the model (shown in the table as ‘treatment effect’). * indicates *p* ≤ 0.05. Abbreviations: MENQoL: Menopause Quality of Life questionnaire; OLE: olive leaf extract.

**Table 3:** HFI questionnaire scores following the OLE and control group in postmenopausal women ^1^.

	OLE			Control			Time × Treatment Interaction	Treatment Effect	Estimated Mean Difference [95% CI]
	Baseline	After 6 Weeks	After 12 Weeks	Baseline	After 6 Weeks	After 12 Weeks			
Sleep Score	1.0 ± 2.1	1.5 ± 2.3	1.9 ± 2.9	1.5 ± 2.8	1.5 ± 2.5	1.0 ± 2.0	*p* = 0.130	*p* = 0.136	+0.5 [−0.1 + 1.2]
Mood Score	0.5 ± 1.0	0.8 ± 1.7	0.7 ± 1.7	1.2 ± 1.9	1.2 ± 2.0	0.9 ± 1.7	*p* = 0.760	*p* = 0.733	+0.1 [−0.5 + 0.7]
Concentration Score	0.7 ± 1.4	0.9 ± 1.9	1.1 ± 2.1	1.1 ± 1.9	1.2 ± 2.3	1.1 ± 1.8	*p* = 0.673	*p* = 0.832	+0.0 [−0.7 + 0.9]

^1^ Values are presented as mean ± SD. *n* = 60. Differences between OLE and control were calculated with a linear mixed model analysis with random intercept, time, and treatment as fixed factors and participant as a random factor. *p*-values for the treatment effect (estimated mean difference [95% CI] between OLE and placebo) were reported. Abbreviations: HFI: Hot Flash Interference scale; OLE: olive leaf extract.

**Table 4:** DEXA scan measurements following the OLE and control group in postmenopausal women ^1^.

	OLE		Control		Treatment Effect Estimated Mean Difference [95% CI]
	Baseline	After 12 Weeks	Baseline	After 12 Weeks	
Body composition					
Total Body FM (g)	24,025 ± 3839	24,241 ± 3577	25,834 ± 6963	25,775 ± 7094	−366 [−909, 177], *p* = 0.182
Est. VAT Mass (g)	384 ± 180	382 ± 165	444 ± 151	436 ± 152	−5.0 [−30, 20], *p* = 0.698
Total Body FFM (g)	45,740 ± 6129	46,164 ± 6319	46,286 ± 4941	46,597 ± 5007	−52.4 [−729, 624], *p* = 0.877
Lean body mass/Height^2^ (kg/m^2^)	15.8 ± 1.3	15.9 ± 1.4	16.2 ± 1.2	16.3 ± 1.3	−0.0 [−0.3, 0.2], *p* = 0.632
ALM index (kg/m^2^)	6.7 ± 0.7	6.7 ± 0.7	6.7 ± 0.7	6.8 ± 0.6	−0.0 [−0.1, 0.0], *p* = 0.662
Bone Mineral Density					
BMD, left arm (g/cm^3^)	0.674 ± 0.053	0.669 ± 0.051	0.689 ± 0.051	0.688 ± 0.056	−0.009 [−0.021, 0.004]; *p* = 0.185
BMD, right arm (g/cm^3^)	0.677 ± 0.048	0.690 ± 0.055	0.695 ± 0.048	0.688 ± 0.055	+0.017 [0.003, 0.030], *p* = 0.019 *
BMD, left ribs (g/cm^3^)	0.615 ± 0.124	0.613 ± 0.125	0.604 ± 0.078	0.600 ± 0.080	−0.002 [−0.026, 0.023], *p* = 0.884
BMD, right ribs (g/cm^3^)	0.576 ± 0.103	0.588 ± 0.131	0.574 ± 0.074	0.559 ± 0.071	0.029 [−0.001, 0.59], *p* = 0.055
BMD, T spine (g/cm^3^)	0.770 ± 0.091	0.759 ± 0.114	0.767 ± 0.081	0.760 ± 0.088	−0.002 [−0.042, 0.038], *p* = 0.928
BMD, L spine (g/cm^3^)	0.930 ± 0.155	0.928 ± 0.138	0.989 ± 0.171	0.979 ± 0.165	−0.002 [−0.026, 0.023], *p* = 0.894
BMD, pelvis (g/cm^3^)	1.102 ± 0.148	1.115 ± 0.150	1.160 ± 0.116	1.165 ± 0.129	0.001 [−0.020, 0.021], *p* = 0.926
BMD, left leg (g/cm^3^)	1.063 ± 0.088	1.067 ± 0.091	1.061 ± 0.077	1.056 ± 0.079	−0.002 [−0.019, 0.015], *p* = 0.830
BMD, right leg (g/cm^3^)	1.058 ± 0.089	1.064 ± 0.095	1.060 ± 0.078	1.059 ± 0.080	−0.005 [−0.025, 0.015], *p* = 0.643
BMD, total body (g/cm^3^)	1.028 ± 0.081	1.037 ± 0.086	1.031 ± 0.079	1.030 ± 0.082	0.004 [−0.006, 0.014], *p* = 0.449
DEXA T-score Total	−1.010 ± 1.060	−0.900 ± 1.109	−0.967 ± 1.041	−0.983 ± 1.083	0.050 [−0.089, 0.189], *p* = 0.476

^1^ Values are presented as mean ± SD; *n* = 57. Differences between OLE and control were calculated with a linear mixed model analysis with random intercept. Treatment was used as a fixed factor, and participant as a random factor. *p*-values for the treatment effect (estimated mean difference [95% CI] between OLE and placebo) were reported. * indicates *p* ≤ 0.05. Abbreviations: ALM: appendicular lean mass; BMD: bone mineral density; Est. VAT: Estimated Visceral Adipose Tissue; FM: fat mass; FFM: fat-free mass; OLE: olive leaf extract; VAT: Visceral Adipose Tissue.

**Table 5:** Handgrip measurements following the OLE and control group in postmenopausal women ^1^.

	OLE			Control			Time × Treatment Interaction	Treatment Effect	Estimated Mean Difference [95% CI]
	Baseline	After 6 Weeks	After 12 Weeks	Baseline	After 6 Weeks	After 12 Weeks			
Higher strength, left hand (kg)	30.2 ± 7.3	30.0 ± 9.0	30.7 ± 7.7	29.6 ± 4.4	29.3 ± 4.8	28.9 ± 4.7	*p* = 0.138	*p* = 0.478	+0.4 [−0.8, 1.7]
Higher strength, right hand (kg)	30.9 ± 7.3	30.1 ± 8.6	30.6 ± 8.4	30.9 ± 5.4	30.9 ± 5.6	30.0 ± 5.8	*p* = 0.055	*p* = 0.597	−0.3 [−1.6, 0.9]

^1^ Values are presented as mean ± SD; *n* = 60. Differences between OLE and control were calculated with a linear mixed model analysis with random intercept. Time × treatment was used as a fixed factor and participant as a random factor. If the interaction term was not significant, it was removed from the model (shown in the table as ‘treatment effect’). Estimated mean difference [95% CI] between OLE and placebo and *p*-values were reported. Abbreviations: OLE: olive leaf extract.

**Table 6:** Blood lipids concentrations following the OLE and placebo intervention period in postmenopausal women ^1^.

	OLE			Control			Time × Treatment Interaction	Treatment Effect	Estimated Mean Difference [95% CI]
	Baseline	After 6 Weeks	After 12 Weeks	Baseline	After 6 Weeks	After 12 Weeks			
TC [mmol/L]	5.72 ± 1.19	5.64 ± 1.19	5.40 ± 0.92	6.02 ± 0.89	6.00 ± 0.73	5.92 ± 0.70	*p* = 0.688	*p* = 0.116	−0.1 [−0.4, 0.0]
HDL [mmol/L]	1.81 ± 0.41	1.80 ± 0.33	1.75 ± 0.33	1.92 ± 0.43	1.87 ± 0.36	1.88 ± 0.42	*p* = 0.414	*p* = 0.530	−0.0 [−0.0, 0.0]
LDL [mmol/L]	3.48 ± 0.96	3.43 ± 0.97	3.24 ± 0.74	3.65 ± 0.80	3.64 ± 0.67	3.57 ± 0.66	*p* = 0.817	*p* = 0.232	−0.1 [−0.3, 0.0]
TG [mmol/L]	0.98 ± 0.31	0.90 ± 0.25	0.93 ± 0.27	1.09 ± 0.47	1.16 ± 0.43	1.15 ± 0.60	*p* = 0.620	*p* = 0.010 *	−0.1 [−0.2, 0.0]
TC/HDL-cholesterol	3.24 ± 0.68	3.16 ± 0.58	3.14 ± 0.58	3.30 ± 0.98	3.34 ± 0.93	3.34 ± 1.07	*p* = 0.983	*p* = 0.095	−0.1 [−0.3, 0.0]
TG/HDL-cholesterol	0.58 ± 0.25	0.52 ± 0.19	0.56 ± 0.21	0.66 ± 0.48	0.69 ± 0.43	0.72 ± 0.62	*p* = 0.912	*p* = 0.029 *	−0.1 [−0.2, −0.0]

^1^ Values are presented as mean ± SD; *n* = 60. Differences between OLE and placebo were calculated with a linear mixed model analysis with random intercept. Differences between OLE and placebo were calculated with a linear mixed model analysis with random intercept. Time × treatment was used as a fixed factor, and participant as a random factor. *p*-values were reported for the treatment effect between OLE and placebo intervention (estimated mean difference [95% CI]). * indicates *p* ≤ 0.05. Abbreviations: TC: total cholesterol; HDL: high-density lipoprotein cholesterol; LDL: low-density lipoprotein cholesterol; TG: triglycerides OLE: olive leaf extract.

## Data Availability

The data in this current study are available from the corresponding author upon reasonable request.

## Supplementary Materials

The following supporting information can be downloaded at: https://www.mdpi.com/article/10.3390/nu16223879/s1, Table S1. IPAQ questionnaire scores following the OLE and placebo intervention period in postmenopausal women. Table S2. DEXA scan measurements following the OLE and placebo intervention period in postmenopausal women. Figure S1. CONSORT study flow diagram.

## Abbreviations

ALMI	Appendicular lean mass index
BMC	Bone mineral density
BMI	Body Mass Index
DXA	Dual-energy X-ray absorptiometry
E2	17-β-oestradiol
ERs	Estrogen receptors
Erα	Estrogen receptor α
Erβ	Estrogen receptor β
FFM	Fat-free mass
FM	Fat mass
HDL-C	High-density lipoproteins
HFI	The Hot Flash Interference scale
IPAQ-L	International Physical Activity Questionnaire long version
LDL-C	Low-density lipoproteins
MENQoL	The Menopause-Specific QoL Questionnaire
MRUM	Metabolic Research Unit Maastricht
OLE	Olive leaf extract
PPARγ	Peroxisome proliferator-activated receptor γ
QoL	Quality of Life
TG	Triacylglycerol
VAT	Visceral abdominal tissue
