# Supplementary material for: Olive Leaf Extract Supplementation Improves Postmenopausal Symptoms: A Randomized, Double-Blind, Placebo-Controlled Parallel Study on Postmenopausal Women
Source: Nutrients. 2024 Nov 14;16(22):3879. doi: 10.3390/nu16223879 (PMC11597182; doi:10.3390/nu16223879)
Supplement: Supplementary file 1 [file nutrients-16-03879-s001.zip › Supplementary Files I.pdf]

SUPPLEMENTARY FILES I

Table S1. IPAQ questionnaire scores following the OLE and placebo intervention period in postmenopausal women[1].

	OLE intervention (n=29)			Placebo (n=31)			Treatment Effect (OLE-Placebo)
	Baseline	After 6 weeks	After 12 weeks	Baseline	After 6 weeks	After 12 weeks	
**IPAQ Total Score**	7144 ± 9350	5535 ± 4652	5837 ± 6930	5502 ± 4042	4378 ± 2376	4667 ± 3787	+5457 [-806+1990], p=0.400
**IPAQ Sitting Score**	269 ± 118	267 ± 109	315 ± 285	282 ± 116	300 ± 125	318 ± 146	-25 [-76+24], p=0.312

[1] Values are means ± SDs. N=60. Differences between OLE and placebo were calculated with a linear mixed model analysis with random intercept. Differences between OLE and placebo were calculated with a linear mixed model analysis with random intercept, time and treatment as fixed factors, and participant as random factors. P-values for the treatment effect (estimated mean difference [95% CI] between OLE and placebo) were reported. Abbreviations: IPAQ: International Physical Activity Questionnaire; OLE: olive leaf extract.

Table S2. DEXA scan measurements following the OLE and placebo intervention period in postmenopausal women[1].

	OLE intervention (n=29)		Placebo (n=31)		Treatment Effect	Treatment Effect (BMI correction)
	Baseline	After 12 weeks	Baseline	After 12 weeks		
Body composition (Fat Mass)						
**Fat mass, left arm (g)**	1533 ± 304	1553 ± 300	1681 ± 480	1686 ± 517	0.382	0.433
**Fat mass, right arm (g)**	1555 ± 304	1584 ± 297	1692 ± 501	1704 ± 537	0.528	0.572
**Fat mass, trunk (g)**	10293 ± 2524	10428 ± 2312	11315 ± 3384	11363 ± 3453	0.586	0.589
**Fat mass, left leg (g)**	4797 ± 798	4756 ± 696	5032 ± 1547	4922 ± 1489	0.054	0.071
**Fat mass, right leg (g)**	4858 ± 913	5081 ± 924	5138 ± 1564	5138 ± 1564	0.679	0.676
**Fat mass, total body (g)**	24025 ± 3839	24241 ± 3577	25834 ± 6963	25775 ± 7094	0.182	0.171
**Fat mass, android (A)(g)**	1749 ± 590	1787 ± 607	2035 ± 763	2081 ± 796	0.454	0.439
**Fat mass, gynoid (G)(g)**	4272 ± 693	4325 ± 661	4769 ± 1331	4723 ± 1352	0.852	0.848

Body composition (Lean + BMC(g))						
**Lean+BMC, left arm (g)**	2177 ± 396	2181 ± 371	2190 ± 318	2221 ± 289	0.601	0.642
**Lean+BMC, right arm (g)**	2400 ± 393	2412 ± 425	2405 ± 363	2428 ± 338	0.700	0.800
**Lean+BMC, trunk (g)**	22432 ± 3780	22572 ± 3965	23506 ± 2674	23613 ± 2574	0.790	0.814
**Lean+BMC, left leg (g)**	7382 ± 1211	7525 ± 1225	7314 ± 979	7412 ± 995	0.852	0.657
**Lean+BMC, right leg (g)**	7574 ± 1235	7691 ± 1223	7536 ± 1018	7598 ± 1023	0.863	0.668
**Lean+BMC, subtotal (g)**	42312 ± 6010	42793 ± 6215	42953 ± 4831	43274 ± 4910	0.962	0.988
**Lean+BMC, head (g)**	3428 ± 285	3371 ± 349	3333 ± 259	3323 ± 256	0.379	0.386
**Lean+BMC, total (g)**	45740 ± 6129	46164 ± 6319	46286 ± 4941	46597 ± 5007	0.877	0.934
**Lean+BMC, android(A)**	3644 ± 645	3825 ± 679	3768 ± 566	3888 ± 541	0.471	0.367
**Lean+BMC, gynoid(G)**	7238 ± 1134	7429 ± 1104	7545 ± 990	7498 ± 936	0.071	0.066
Body composition (Total mass (g))						
**Total Mass, left arm (g)**	3711 ±580	3772 ±592	3871 ± 623	3907 ± 667	0.753	0.866
**Total Mass, right arm (g)**	3921 ±620	3996 ±611	4098 ± 707	4133 ± 750	0.903	0.993
**Total Mass, trunk (g)**	33070 ±4845	33370 ±5063	34822 ± 5557	34973 ± 5586	0.821	0.833
**Total Mass, left leg (g)**	12183 ±1620	12284 ±1552	12346 ± 2290	12335 ± 2299	0.117	0.063
**Total Mass, right leg (g)**	12424 ±1776	12611 ±1651	12675 ± 2371	12703 ± 2343	0.260	0.165
**Total Mass, subtotal (g)**	65355 ±8648	66035 ± 8771	67813 ± 10819	68056 ± 11079	0.389	0.373
**Total Mass, head (g)**	4410 ±351	4370 ±318	4306 ± 308	4315 ± 300	0.436	0.430
**Total Mass, total body (g)**	69766 ± 8773	70406 ± 8900	72120 ± 10888	72039 ± 11546	0.934	0.963
**Total Mass, android (A) (g)**	5393 ±1120	5613 ±1210	5804 ± 1247	5969 ± 1259	0.892	0.910
**Total Mass, gynoid(G) (g)**	11510 ±1558	11791 ±1428	12315 ± 2152	12293 ± 2216	0.164	0.167
Body composition (% Fat))						
**Fat, left arm (%)**	41.3 ± 5.1	41.3 ± 5.1	42.8 ± 6.8	42.4 ± 6.6	0.347	0.499
**Fat, right arm (%)**	39.3 ± 4.6	39.7 ± 4.8	40.7 ± 6.5	40.4 ± 6.6	0.998	0.989
**Fat, trunk (%)**	30.8 ± 4.4	31.0 ± 3.5	31.8 ± 5.3	31.7 ± 5.6	0.774	0.851
**Fat, left leg (%)**	39.4 ± 5.0	38.8 ± 4.6	40.0 ± 5.8	39.2 ± 5.4	0.084	0.136
**Fat, right leg (%)**	39.0 ± 5.2	39.1 ± 4.2	39.8 ± 5.7	39.5 ± 5.3	0.199	0.313
**Fat, subtotal (%)**	35.2 ± 3.4	35.2 ± 3.0	36.0 ± 5.0	35.7 ± 5.1	0.278	0.342

**Fat, head (%)**	22.7 ± 1.0	22.8 ± 1.1	22.6 ± 1.1	23.0 ± 1.0	0.767	0.770
**Fat, total body (%)**	34.0 ± 3.2	34.4 ± 2.9	35.3 ± 4.8	35.0 ± 4.9	0.915	0.824
**Fat, android(A) (%)**	31.7 ± 5.8	31.0 ± 5.5	34.0 ± 6.6	33.6 ± 7.0	0.097	0.103
**Fat, gynoid(G) (%)**	37.1 ± 4.3	37.1 ± 3.8	38.1 ± 4.8	37.8 ± 4.8	0.363	0.442
Adipose Indices Results						
**Total Body %Fat**	34.4 ± 3.3	34.4 ± 2.9	35.3 ± 4.8	35.0 ± 4.9	0.327	0.392
**Fat Mass/Height² (Kg/m²)**	8.7 ± 1.2	8.7 ± 1.0	9.4 ± 2.4	9.4 ± 2.4	0.105	0.109
**Android/Gynoid Ratio**	0.8 ± 0.1	0.8 ± 0.1	0.8 ± 0.1	0.8 ± 0.1	0.321	0.355
**%Fat Trunk/%Fat Legs**	0.8 ± 0.1	0.8 ± 0.1	0.8 ± 0.1	0.8 ± 0.1	0.653	0.661
**Trunk /Limb Fat Mass Ratio**	0.8 ± 0.2	0.8 ± 0.1	0.8 ± 0.1	0.8 ± 0.1	0.799	0.819
**Est. VAT Mass (g)**	384.3 ± 180.4	381.6 ± 165.1	443.5 ± 150.7	436.0 ± 152.0	0.698	0.735
**Est. VAT Volume (cm³)**	415.5 ± 195.0	412.4 ± 178.5	479.5 ± 162.9	471.3 ± 164.3	0.692	0.728
**Est. VAT Area (cm²)**	79.7 ± 37.4	79.1 ± 43.2	97.9 ± 50.2	90.4 ± 31.5	0.575	0.589
Adipose Indices (T-score)						
**T-score Total Body %Fat**	0.2 ± 0.4	0.2 ± 0.3	0.3 ± 0.6	0.2 ± 0.6	0.314	0.367
**T-score Fat Mass/Height² (Kg/m²)**	0.1 ± 0.3	0.1 ± 0.2	0.2 ± 0.5	0.2 ± 0.5	0.253	0.278
**T-score %Fat Trunk/%Fat Legs**	0.2 ± 0.8	0.2 ± 0.5	0.2 ± 0.6	0.3 ± 0.6	0.513	0.541
**T-score Trunk /Limb Fat Mass Ratio**	0.1 ± 0.9	0.1 ± 0.7	0.3 ± 0.7	0.3 ± 0.7	0.884	0.927
Lean Indices results						
**Lean /Height² (kg/m²)**	15.8 ± 1.3	15.9 ± 1.4	16.2 ± 1.2	16.3 ± 1.3	0.632	0.692
**Appen.Lean/Height² (Kg/m²) (ALMindex)**	6.7 ± 0.7	6.7 ± 0.7	6.7 ± 0.7	6.8 ± 0.6	0.662	0.767
Lean Indices (T-Score)						
**T-score Lean /Height² (kg/m²)**	-0.10 ± 0.6	-0.06 ± 0.6	0.06 ± 0.6	0.09 ± 0.6	0.779	0.846
**T-score Appen.Lean/Height² (Kg/m²)**	-0.18 ± 0.6	-0.09 ± 0.6	-0.11 ± 0.6	-0.06 ± 0.6	0.844	0.965
DXA Summary (Bone Mineral Density)						
**BMD, left arm (g/cm³)**	0.674 ± 0.053	0.669 ± 0.051	0.689 ± 0.051	0.688 ± 0.056	0.185	0.189
**BMD, right arm (g/cm³)**	0.677 ± 0.048	0.690 ± 0.055	0.695 ± 0.048	0.688 ± 0.055	0.019*	0.015*
**BMD, left ribs (g/cm³)**	0.615 ± 0.124	0.613 ± 0.125	0.604 ± 0.078	0.600 ± 0.080	0.884	0.989
**BMD, right ribs (g/cm³)**	0.576 ± 0.103	0.588 ± 0.131	0.574 ± 0.074	0.559 ± 0.071	0.055	0.076

**BMD, T spine (g/cm³)**	0.770 ± 0.091	0.759 ± 0.114	0.767 ± 0.081	0.760 ± 0.088	0.928	0.898
**BMD, L spine (g/cm³)**	0.930 ± 0.155	0.928 ± 0.138	0.989 ± 0.171	0.979 ± 0.165	0.894	0.841
**BMD, pelvis (g/cm³)**	1.102 ± 0.148	1.115 ± 0.150	1.160 ± 0.116	1.165 ± 0.129	0.926	0.921
**BMD, left leg (g/cm³)**	1.063 ± 0.088	1.067 ± 0.091	1.061 ± 0.077	1.056 ± 0.079	0.830	0.917
**BMD, right leg (g/cm³)**	1.058 ± 0.089	1.064 ± 0.095	1.060 ± 0.078	1.059 ± 0.080	0.643	0.834
**BMD, subtotal (g/cm³)**	0.884 ± 0.075	0.889 ± 0.081	0.892 ± 0.066	0.889 ± 0.067	0.673	0.529
**BMD, total body (g/cm³)**	1.028 ± 0.081	1.037 ± 0.086	1.031 ± 0.079	1.030 ± 0.082	0.449	0.350
DXA Results Total						
**DEXA T-score Total**	-1.010 ± 1.060	-0.900 ± 1.109	-0.967 ± 1.041	-0.983 ± 1.083	0.476	0.396
**DEXA PR (%) Total**	93.068 ± 7.372	93.851 ±7.798	93.290 ± 7.290	93.200 ± 7.334	0.398	0.297

1 Values are means ± SDs; n=57. Differences between OLE and placebo were calculated with a linear mixed model analysis with random intercept. Treatment was used as a fixed factor, and participant as a random factor. P represents the p-values for the difference in estimated means after 12 weeks of intervention between OLE and placebo corrected for baseline differences and BMI. *Indicates $p \leq 0.05$. Abbreviations: ALM: Appendicular lean mass; ALMI: ALM Index; Android=abdomen region; BMC: Bone Mineral Content; BMD: Bone Mineral Density; Est. VAT: Estimated Visceral Adipose Tissue; OLE: olive leaf extract; PR= percentage; Total mass: FM + Lean Mass + BMC; VAT: Visceral Adipose Tissue.
