# Supplementary figures and images for: Olive Leaf Extract Supplementation Improves Postmenopausal Symptoms: A Randomized, Double-Blind, Placebo-Controlled Parallel Study on Postmenopausal Women
Source: Nutrients. 2024 Nov 14;16(22):3879. doi: 10.3390/nu16223879 (PMC11597182; doi:10.3390/nu16223879)

## SUPPLEMENTARY FILES II

**Figure S1.** CONSORT study flow diagram.

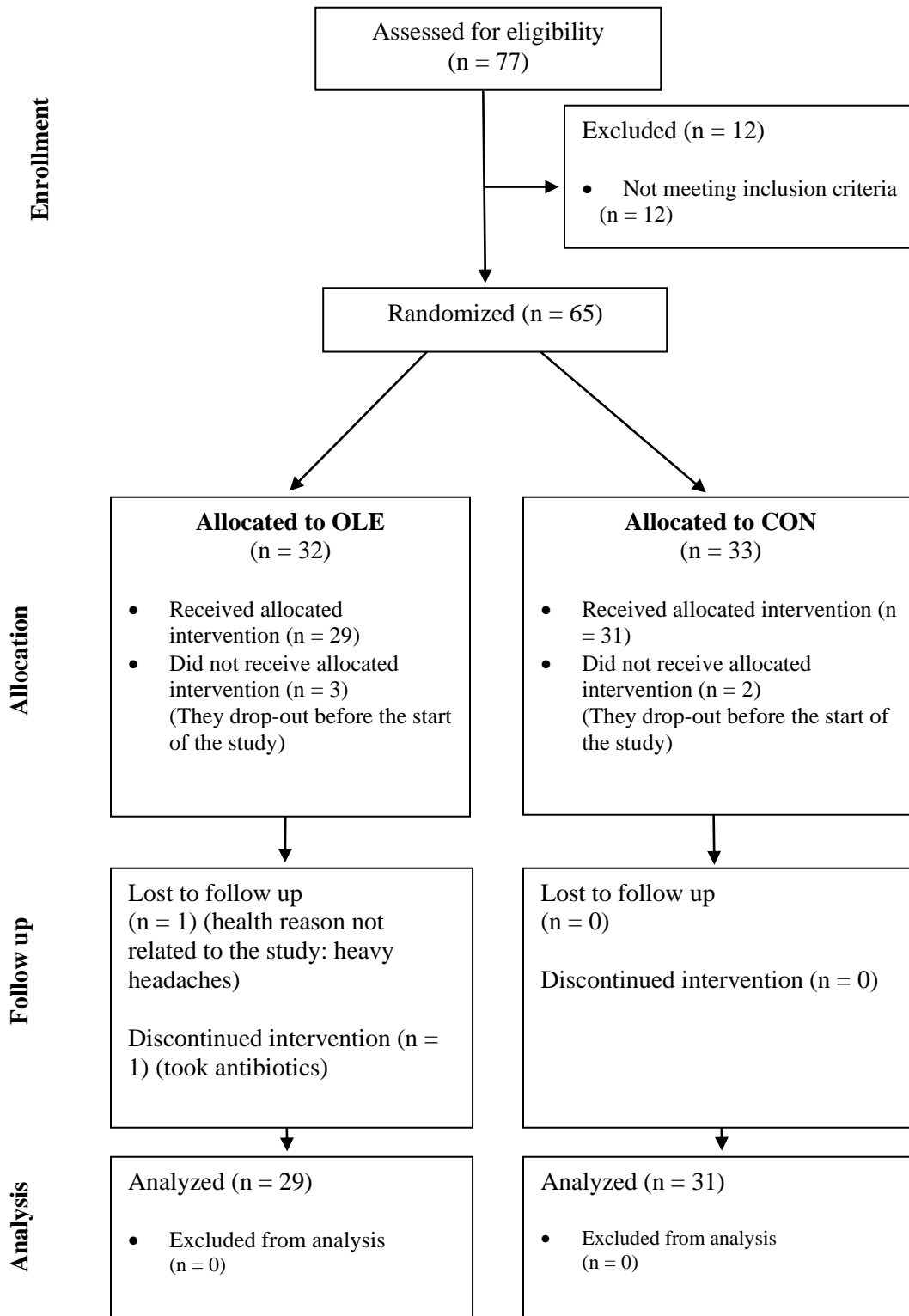

Supplement: Supplementary file 1 [file nutrients-16-03879-s001.zip › Supplementary Files II.pdf]
